# PD56 Long-Term Use Of Lisdexamfetamine For Attention-Deficit/Hyperactivity Disorder: What Does The Evidence Say?

**DOI:** 10.1017/S0266462324003143

**Published:** 2025-01-07

**Authors:** Maíra Catharina Ramos, Flávia Tavares Silva Elias

## Abstract

**Introduction:**

Attention-deficit/hyperactivity disorder (ADHD) is a neuropsychiatric disorder that can interfere with school and academic life, work, and even personal relationships. One of the alternative medications is lisdexamfetamine (LDX), a prodrug amphetamine preparation that lasts an average of 13 hours due to its gradual conversion. Since LDX is used continuously, it is necessary to evaluate its long-term efficacy and safety.

**Methods:**

A rapid health technology assessment (HTA) was performed. Searches were conducted in the PubMed, Embase, Web of Science, and Cochrane Library databases using descriptors and their respective synonyms to identify studies on the long-term efficacy and safety of LDX in people with ADHD. Interventional and control group studies with a follow-up period of more than five weeks were included. Secondary studies were excluded. The reference lists of included studies were screened to identify potentially eligible publications that were not found in the database searches. Study selection was carried out in two stages, with screening of titles and abstracts and then assessment of full-text articles for eligibility.

**Results:**

This rapid HTA included 32 studies. The population included patients aged five to 55 years, and the longest follow-up was 108 weeks. In general, the literature reported a decrease in symptoms in the first five to six weeks of treatment, stabilizing thereafter. After 108 weeks, the mean change in ADHD Rating Scale-IV hyperactivity/impulsivity was -25.8 (95% confidence interval [CI]: -27.0, -24.5; p<0.001) and -13.1 (95% CI: -13.8, -12.4; p< 0.001) for the ADHD Rating Scale-IV inattention subscale. However, psychiatric disorder system organ class adverse events were frequent, including irritability, anxiety, and aggression, in addition to suicide attempts in severe cases.

**Conclusions:**

It appears that long-term use of LDX has been associated with good clinical results in the treatment of ADHD, with treatment effectiveness remaining stable during the time observed (two-year follow-up). However, adverse events, especially psychiatric disorders, require attention as they can be confused with symptoms of the disease.

